# Dl-3-n-butylphthalide attenuates DOX-induced cardiotoxicity in mice by inhibiting Nrf2/Keap1 complex formation

**DOI:** 10.3389/fphar.2025.1542296

**Published:** 2025-04-29

**Authors:** Yixiao Yan, Mingzhen Fang, Cong Zhao, Xinru Lin, Chen Tong, Cheng Xiang, Ya Ran, Xuelian Wang, Shuixin Li, Gaozhi Chen, Lili Fu

**Affiliations:** ^1^ Oujiang Laboratory (Zhejiang Lab for Regenerative Medicine, Vision and Brain Health), School of Pharmaceutical Sciences, Wenzhou Medical University, Wenzhou, Zhejiang, China; ^2^ Cixi Biomedical Research Institute, Wenzhou Medical University, Wenzhou, Zhejiang, China

**Keywords:** drug-induced cardiotoxicity (DICT), doxorubicin (DOX), nuclear factor erythroid 2-related factor 2 (Nrf2), Dl-3-n-butylphthalide (NBP), kelch-like ECH associated protein-1 (Keap1), C57BL/6 mice

## Abstract

**Introduction:**

Drug-induced cardiotoxicity (DICT), defined as myocardial injury caused by direct or indirect toxicity of therapeutic agents, disrupts cardiovascular homeostasis, underscoring the urgent need for preventive strategies in clinical practice. Doxorubicin (DOX), a clinically established anthracycline chemotherapeutic, induces dose-dependent cardiotoxicity driven by reactive oxygen species overproduction. Notably, Dl-3-n-butylphthalide (NBP), a bioactive phytochemical derived from celery, has shown potential in mitigating DOX-induced cardiomyopathy via its antioxidant activity. Therefore, this study aimed to investigate the protective effects of NBP on DOX-induced cardiomyopathy, with a focus on elucidating the underlying mechanisms.

**Method:**

We developed both *in vivo* and *in vitro* models of DOX-induced cardiotoxicity. For the animal model, male C57BL/6 mice were administered with DOX (4 mg/kg, i.p.) once a week for 3 weeks. For the cell model, H9C2 myoblasts were exposed to 1 μM DOX for at least 6 h to establish acute cardiotoxicity.

**Results:**

Our results demonstrate that NBP significantly improves cardiac function, as evidenced by approximately 10% increase in cardiac functional parameters (ejection fraction and left ventricular shortening fraction). Besides, NBP exerts favorable effects on cardiac inflammation, apoptosis, fibrosis, and mitochondrial damage both *in vivo* and *in vitro*. Further mechanistic investigations revealed that NBP blocks the interaction between Kelch-like ECH-associated protein-1 (Keap1) and Nrf2, thereby preventing the formation of the Nrf2/Keap1 complex.

**Discussion:**

This study indicate that NBP alleviates DOX-induced cardiotoxicity by inhibiting Nrf2/Keap1 complex formation, highlighting its potential as a therapeutic agent for DICT and suggest that Nrf2/Keap1 may be a potential therapeutic target for the management of this condition.

## 1 Introduction

Cardiovascular diseases (CVDs), including drug-induced cardiotoxicity (DICT), are the major cause of mortality worldwide (Global burden of 369 diseases and injuries in 204 countries and territories, 1990-2019: a systematic analysis for the Global Burden of Disease Study, 2019, 2020), the death rate of which is expected to reach more than 20 million by 2030 (Raghu et al., 2015). DICT is caused by toxic side effects, interactions and/or improper application of drugs. Various drugs, including antiarrhythmics, sympathomimetics, penicillin, and imidazole, exhibit mild and negligible cardiotoxicity within therapeutic doses. However, doxorubicin (DOX), a chemotherapeutic agent for multiple cancers (Joshi et al., 2021; Lin et al., 2023; Tacar et al., 2013), is associated with severe cardiotoxicity, potentially leading to congestive heart failure and poor prognosis (Briehl, 2015).

During therapy, DOX undergoes redox reactions, converting its quinone structure into semiquinone free radicals (one-electron reduction) and hydroquinone (two-electron reduction), leading to excessive reactive oxygen species (ROS) production (Li et al., 2021; Ma et al., 2013). While ROS generation can induce tumor cell apoptosis or necrosis, it also damages normal cells, particularly ROS-sensitive cardiomyocytes, causing irreversible injury and limiting the clinical use of DOX. Given the role of ROS in apoptosis (Benhar, 2020; Bian et al., 2019) and inflammation (Morris et al., 2022), antioxidant strategies are considered a promising approach to mitigate DOX-induced cardiotoxicity.

Dl-3-n-butylphthalide (NBP) belonging to the simple phthalide class is a natural product extracted from celery and has been granted approval by the State Food and Drug Administration (SFDA) of China as a means of clinical intervention of this disease (Wang et al., 2018). Previous studies have indicated that the pharmacological activity of NBP is associated with multiple bioactivities (Li et al., 2019; Que et al., 2021; Zhang et al., 2022), and we specifically focused on antioxidant activity because of the important role of ROS. However, the specific molecular mechanism of NBP remains unclear. In this study, on the basis of the properties of NBP as well as its potential protective effect on the heart, we aimed to explore whether NBP could alleviate myocardial injury induced by DOX and its underlying mechanism. To this end, we developed an animal model by dissolving 4 mg/kg DOX in normal saline and injecting it intraperitoneally into C57BL/6 mice once a week for 3 weeks. H9C2 cardiomyocytes were subsequently used to establish a cell model of DOX-induced cardiotoxicity. We validated both *in vivo* and *in vitro* that NBP could exert cardioprotective effects on DOX-induced inflammation, apoptosis, fibrosis, oxidative stress and mitochondrial damage. We further determined the molecular mechanism of NBP. Through the effective inhibition of the interaction between Kelch-like ECH-associated protein-1 (Keap1) and Nrf2, NBP attenuates the ubiquitination-dependent proteolytic degradation of the Nrf2 protein. Consequently, it manifests its antioxidant efficacy in mitigating DOX-induced cardiotoxicity.

## 2 Materials and methods

### 2.1 Chemicals and reagents

Doxorubicin (DOX) was acquired from Solarbio Science & Technology Co., Ltd. (D8740, Solarbio, Beijing, China). High-purity NBP (99.97%) was obtained from the National Institutes for Food and Drug Control (101035, NIFDC, Beijing, China) and was composed with dimethyl sulfoxide (DMSO) (D8371, Solarbio, Beijing, China) for cell experiments. NBP normal saline injection was supplied by CSPC Pharmaceutical Group Limited (Shijiazhuang, Hebei, China) for animal experiments. Topscience (Shanghai, China) supplied ML385 (T4360), and MedChemExpress (Shanghai, China) supplied MG132 (HY-13259).

Antibodies were obtained from these suppliers: anti-Nrf2 (66504-1-Ig, 16396-1-AP), anti-AMPK alpha (10929-2-AP), anti-HO-1/HMOX1 (10701-1-AP), anti-alpha tubulin (66031-1-Ig), anti-collagen type I (67288-1-Ig), anti-TGF beta 1 (21898-1-AP), anti-smooth muscle actin (14395-1-AP), anti-Bcl2 (26593-1-AP), anti-caspase 3/P17/P19 (19677-1-AP), and anti-KEAP1 (10503-2-AP) were obtained from Proteintech (Wuhan, China). Anti-phospho-AMPK alpha (T55608S) was obtained from Abmart (Shanghai, China). Anti-Bax (ET1603-34) and anti-NQO1 (ET1702-50) antibodies were obtained from HUABIO (Hangzhou, China).

Mouse TNF-α (BMS607-3TEN) and IL-6 (KMC0061) ELISA kits were acquired from Invitrogen (Carlsbad, CA, USA).

### 2.2 Compound solution preparation

The NBP compound was dissolved in DMSO to achieve a stock concentration of 80 mM and then stored at −20°C for long-term preservation. For *in vitro* experiments, the NBP stock solution was further diluted with DMSO to obtain working solutions of 20 mM and 40 mM. According to the manufacturer’s instructions, ML385 and MG132 were dissolved in DMSO to final concentrations of 4 mM and 20 mM, respectively, and both were stored at −20°C. In all cell-based assays, the concentration of DMSO was maintained at a maximum of 0.1% (v/v).

For *in vitro* experiments, DOX was dissolved in normal saline to achieve a concentration of 2 mg/mL and subsequently filtered through a 0.22-micron membrane. For *in vivo* experiments, DOX was dissolved in double-distilled water (ddH2O) to a concentration of 1 mM, followed by filtration through a 0.22-μm membrane. ML385 was dissolved sequentially in DMSO, polyethylene glycol 300 (PEG300), and physiological saline (0.9% NaCl) at volume ratios of 10%, 45%, and 45%, respectively. The final working solution was adjusted to a concentration of 3 mg/kg for *in vivo* delivery. Both solutions were stored under appropriate conditions and used immediately or as required for experimental procedures.

### 2.3 Animal study

C57BL/6 male mice (8–10 weeks) weighing 18–22 g were sourced from the Animal Centre at Wenzhou Medical University (Wenzhou, China). The mice were raised under controlled environmental conditions, including a 12-hour light/dark cycle, ambient temperature maintained at 20°C–25°C, and relative humidity of 40%–60%. Standard rodent chow and water were provided *ad libitum* throughout the acclimatization and experimental periods. All animal care and experimental procedures were approved by the Wenzhou Medical College Animal Policy and Welfare Committee (No. wydw2021-0437).

In the first set of experiments about the pharmacological activity of NBP, a total of 32 mice were randomly allocated into four experimental groups (n = 8 per group): (i) the control group; (ii) the DOX group; (iii) the 25 mg/kg NBP-treated DOX group; and (iv) the 50 mg/kg NBP-treated DOX group. After 1 week of acclimation, the ii, iii and iv groups were administered with DOX (4 mg/kg, i.p.) once a week for 3 weeks (Li et al., 2018), and the iii and iv groups were administered with NBP every other day for 3 weeks (25 mg/kg or 50 mg/kg, respectively, i.p.) after the first injection of DOX (Wang et al., 2021). Before being euthanized under anesthesia, all mice in each group underwent transthoracic echocardiography to assess cardiac function. Following the procedure, blood samples were collected via cardiac puncture for serum isolation, and heart tissues were rapidly excised, snap-frozen in liquid nitrogen, and stored at −80°C for subsequent biochemical and molecular analyses.

To further investigate the mechanism of NBP, we conducted a second experiment. A total of 32 mice were randomly allocated into four experimental groups (n = 8 per group): (i) the control group; (ii) the DOX group; (iii) the 50 mg/kg NBP-treated DOX group; and (iv) the 50 mg/kg NBP-30 mg/kg ML385-treated DOX group. After 1 week of acclimation, the ii, iii and iv groups were administered with DOX (4 mg/kg, i.p.) once a week for 3 weeks, and the ⅲ group was administered with NBP every other day for 3 weeks (50 mg/kg, i.p.) after the first injection of DOX. The ⅳ group was received a combined therapy of NBP (50 mg/kg, i.p.) and ML385 (30 mg/kg, i.p.) (Wu et al., 2022) administered every other day for 3 weeks to verify the potential mechanism of NBP.

### 2.4 Western blot analysis

Extraction of proteins from heart tissue or H9C2 cells was performed using a lysis buffer (AR0101/0103, Boster Biological Technology Co. Ltd., Pleasanton, CA, USA), and the protein concentrations were subsequently measured via Coomassie brilliant blue (5000205, Bio-Rad, Hercules, CA, USA). By SDS-PAGE, the proteins were separated and subsequently electrotransferred to PVDF membranes (IPVH00010, Sigma, St. Louis, MO, USA). After blocking with 5% milk for 1 h at room temperature, the PVDF membranes were exposed to primary antibodies overnight (4°C), and then incubated with the corresponding secondary antibodies for another 1 h. Finally, the membranes were imaged via a chemiluminescence gel imaging system.

### 2.5 Real-time quantitative polymerase chain reaction (qPCR)

Extraction of total RNA from heart tissues or H9C2 cells was performed using TRIzol (Takara Bio, Shiga, Japan). Vazyme (Nanjing, China) provided the HiScript III All-in-one RT SuperMix Perfect for qPCR (R333-01) of reverse transcription and ChamQ Universal SYBR qPCR Master Mix (Q711-03) for quantitative PCR. The sequences of primers provided by Tsingke (Beijing, China) are listed in Supplementary Table S1.

### 2.6 Analysis of serological markers

Whole blood samples were collected from anaesthetized mice and centrifuged at 3,000 rpm for 15 min at 4°C to isolate serum. The serum was subsequently stored at −80°C until further analysis. The concentrations of creatine kinase MB isoenzyme (CK-MB) and lactic dehydrogenase (LDH) were quantified using commercially available assay kits (CK-MB: E006-1-1; LDH: A020-2-2), which were obtained from Nanjing Jiancheng BioTech (Nanjing, China).

The assay of serum samples for mouse tumour necrosis factor-alpha (TNF-α) and interleukin-6 (IL-6) was conducted in accordance with the manufacturer’s instructions. The protein concentrations were determined using the standard curve approach.

### 2.7 Paraffin embedding and sectioning

Heart tissues were fixed in 4% formalin for 24 h and subsequently embedded in paraffin. The embedded tissues were sectioned into 5 μm slices and further subjected to a series of staining protocols, including H&E staining, Masson’s trichrome staining, immunohistochemical staining, and terminal deoxynucleotidyl transferase dUTP nick-end labeling (TUNEL) staining.

### 2.8 Frozen embedding and sectioning

Fresh heart tissues were embedded in Neg-50 Frozen Section Medium (6502, Thermo Fisher Scientific, Waltham, MA, USA) and rapidly frozen at −20°C. The frozen blocks were stored at −80°C until sectioning. For sectioning, the tissue blocks were equilibrated to −20°C in a cryostat (NX50, Thermo Fisher Scientific, Waltham, MA, USA), and serial sections of 5 μm thickness were cut. The sections were mounted onto glass slides, air-dried, and stored at −80°C for further analysis.

### 2.9 Immunohistochemical determination

Following rehydration, the sections for immunohistochemistry underwent antigen retrieval in a 0.01 mol/L citrate buffer (pH 6.0) via water bath heating. They were then incubated in 3% hydrogen peroxide in methanol for 30 min at RT. After being blocked with 5% BSA (A8020, Solarbio, Beijing, China), the slides were incubated with anti-F4/80 (1:200), anti-α-SMA (1:200) or anti-Bax (1:200) antibodies at 4°C overnight. Subsequently, the sections were exposed to the respective secondary antibody at a dilution of 1:200 for 1 h. The immune reaction was detected by DAB (ZLI-9018, ZSGB-BIO, Beijing, China) and the nuclei were counterstained by hematoxylin. Images were acquired via a Nikon microscope equipped with a digital camera (Tokyo, Japan).

### 2.10 Terminal deoxynucleotidyl transferase dUTP nick-end labeling (TUNEL) staining

The TUNEL assay was conducted following the guidelines provided by the manufacturer for the One Step TUNEL Apoptosis Assay Kit (C1090, Beyotime, Shanghai, China). After deparaffinization and rehydration, the heart tissue sections were treated with Proteinase K (20 μg/mL in PBS) for 30 min at 37°C to enhance permeability. After washed twice with PBS, the sections were incubated with the TUNEL reaction mixture at 37°C for 60 min in the dark. Following three PBS washes, the sections were coverslipped with DAPI (S2110, Solarbio, Beijing, China). The stained sections were observed under a fluorescence microscope.

### 2.11 Cell culture and treatment

H9C2 myoblasts were obtained from the Cell Bank of the Chinese Academy of Sciences (Shanghai, China). The cells were cultured in DMEM that was enriched with 10% (v/v) FBS, 100 U/mL penicillin G, and 100 mg/mL streptomycin. The cells were kept at a temperature of 37°C in an incubator that maintained a humidified environment containing 5% CO_2_.

Stock solutions of 1 mM DOX in ddH_2_O were melted on ice to obtain the complexes. The mixtures were then diluted into a serum-containing cell culture medium to achieve a final concentration of 1 μM (Li et al., 2016; Octavia et al., 2012). H9C2 cells were pretreated with NBP at a concentration of 20 or 40 μM for 1 h, and subsequently exposed to DOX for 6 h, 12 h or 24 h to conduct follow-up experiments.

### 2.12 Transmission electron microscopy (TEM) staining

Fresh cardiac tissue blocks were carefully dissected using a sharp blade and immediately fixed in TEM fixative at 4°C. Subsequently, the tissues were post-fixed with 1% osmium tetroxide (OsO4) in 0.1 M phosphate buffer (PB, pH 7.4) for 2 h at room temperature. After removal of OsO4, the tissues were thoroughly rinsed in 0.1 M PB (pH 7.4) three times, 15 min each. Dehydration was performed at room temperature using a graded ethanol series: 30% ethanol (20 min), 50% ethanol (20 min), 70% ethanol (20 min), 80% ethanol (20 min), 95% ethanol (20 min), 100% ethanol (twice, 20 min), and 100% acetone (15 min). Following dehydration, the tissues underwent resin infiltration and embedding as follows: a 1:1 mixture of acetone and EMBed 812 resin (2–4 h, 37°C), a 1:2 mixture of acetone and EMBed 812 resin (overnight, 37°C), and pure EMBed 812 resin (5–8 h, 37°C). The tissues were then transferred into embedding molds filled with pure EMBed 812 resin and incubated at 37°C overnight. The molds were subsequently moved to a 60°C oven for polymerization for more than 48 h. After polymerization, the resin blocks were removed from the molds for further processing. Ultrathin sections (60–80 nm) were cut using an ultramicrotome and collected onto 150-mesh copper grids coated with formvar film for subsequent staining and TEM observation.

### 2.13 JC-1 staining

JC-1 staining was performed in accordance with the manufacturer’s instructions for the Mitochondrial Membrane Potential Detection Kit (JC-1) (C2003, Beyotime, Shanghai, China). Briefly, cells were seeded in 6-well plates and cultured until they reached 70%–80% confluency. The cells were then incubated with JC-1 staining solution at 37°C for 20 min in the dark. Following incubation, the cells were washed twice with PBS to remove unbound dye. Mitochondrial membrane potential was assessed by visualizing the cells under a fluorescence microscope. Red fluorescence indicated the presence of JC-1 aggregates in mitochondria with intact membrane potential, while green fluorescence represented JC-1 monomers in depolarized mitochondria.

### 2.14 Reactive oxygen species (ROS) and glutathione measurement

Dihydroethidium (DHE) staining was conducted following the guidelines provided by the manufacturer for the ROS assay kit for superoxide anion with DHE (S0063, Beyotime, Shanghai, China). For H9C2 myoblasts, cells were seeded in 6-well plates and cultured until they reached 70%–80% confluency. Cells were then incubated with 10 μM DHE at 37°C for 20 min in the dark, washed twice with PBS, and imaged using fluorescence microscopy. For heart tissue analysis, frozen sections were equilibrated at room temperature for 5 min, rinsed with distilled water, and incubated with 10 μM DHE at 37°C for 1 h in the dark. Sections were washed three times with PBS, mounted with DAPI medium (S2110, Solarbio, Beijing, China), and analyzed by fluorescence microscopy. The GSH and GSSG Levels of heart tissues were measured using a GSH and GSSG Assay Kit (S0053, Beyotime, China).

### 2.15 ATP assay

In accordance with the manufacturer’s protocol, the ATP levels of H9C2 myoblasts were quantified by an ATP assay kit (S0026, Beyotime, Shanghai, China). By applying the measured luminescence values to a standard curve, the ATP concentration in each cell lysate sample was determined.

### 2.16 Proximity ligation assay (PLA)

The cells were seeded in 12-well plates covered with glass, and proximity ligation assays were carried out after the cell density reached 70%–80%. After drug treatment, the cells were fixed with 4% paraformaldehyde for 30 min, followed by permeabilization with 0.1% Triton X-100 for 20 min. The cells were transferred onto slides and fixed with nail polish. In accordance with the manufacturer’s protocol, the samples were blocked with blocking solution for 60 min in a 37°C incubator and incubated with a mixture of anti-Nrf2 and anti-Keap1 primary antibodies in a humidified chamber at 4°C overnight. The samples were sequentially incubated with PLUS and MINUS PLA Probes (60 min, 37°C), ligase solution (45 min, 37°C) and polymerase solution (100 min, 37°C). For the incubation of different solutions, the samples were washed with buffer (provided with the kit). After the oligonucleotides on the PLA probe were ligated and amplified, the slides were mounted with a coverslip using a minimal volume of Duolink PLA Mounting Medium with DAPI. Finally, the samples were viewed via a confocal laser scanning microscope (Nikon, Tokyo, Japan).

### 2.17 Co-immunoprecipitation (Co-IP) assay

The cells were seeded in 60 mm dishes, and immunoprecipitation assays were carried out after the cell density reached 70%–80%. The cells were collected and lysed using lysis buffer provided by Boster Biological Technology Co. Ltd. (AR0101/0103, Pleasanton, CA, USA). The lysates were then centrifuged at 12,000 rpm for 10 min at a temperature of 4°C to isolate the supernatant. A total of 1.5 μL of antibody was added to 800 μg of protein, and the mixture was gently agitated at a temperature of 4°C overnight. The immune complexes were collected with protein A+ G agarose beads provided by Beyotime (P2012, Shanghai, China), and the precipitates were washed three times with precooled PBS. After adding adequate protein loading buffer, the prewashed agarose beads were heated in a metal bath at a temperature of 100°C for 10 min.

### 2.18 Statistical analysis

All data were collected from at least three independent replicates and analyzed using GraphPad Prism 9.0 software. The results are expressed as the means ± standard deviations (means ± SDs). The normality of the data distribution was assessed using the Shapiro-Wilk test. Based on the normality test results, statistical significance was determined using either one-way analysis of variance (ANOVA) or the Kruskal-Wallis test. A *P*-value <0.05 (* or ^#^ *P* < 0.05, ^##^ or **P < 0.01 and ^###^ or ***P < 0.001) was considered significant.

## 3 Results

### 3.1 Protective effects of NBP on DOX-induced cardiotoxicity

**FIGURE 1:**
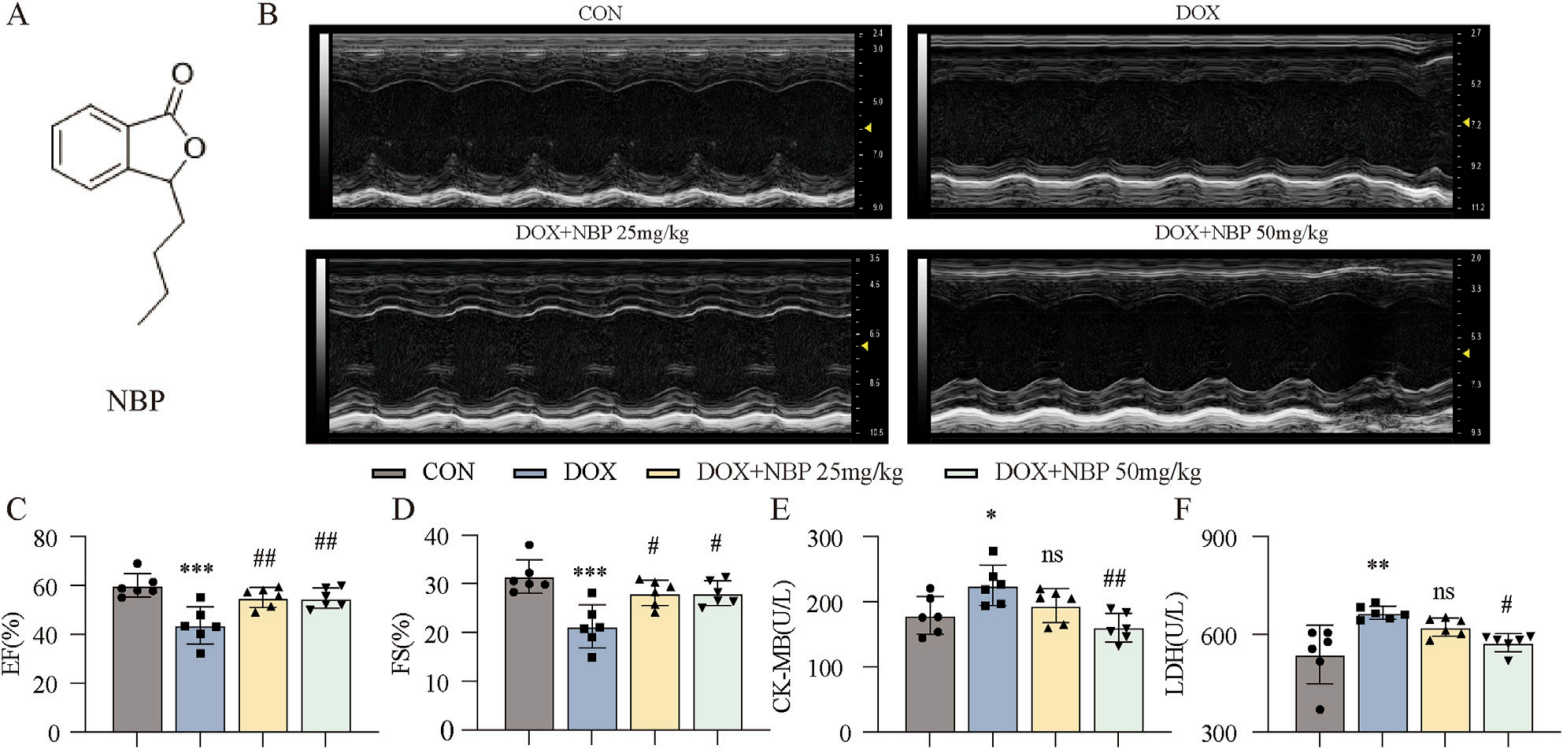
Therapeutic effects of NBP on cardiac function in DOX-induced cardiomyopathy mice. **(A)** The molecular structure of NBP was shown. **(B)** Representative cardiac ultrasound images of each group were obtained. **(C,D)** Quantitative analysis of cardiac functional parameters, including ejection fraction (EF%) and left ventricular shortening fraction (FS%), was performed using Vevo Lab software (n = 6 per group). **(E,F)** Quantitative analysis of serum cardiac biomarkers was performed. The concentrations of creatine kinase-isoenzyme (CK-MB) and lactate dehydrogenase (LDH) in murine serum samples were determined using dedicated kits (n = 6 per group). The data are presented as the means±SDs. Different groups were compared with a one-way analysis of variance (ANOVA). *P < 0.05, **P < 0.01, ***P < 0.001 compared with CON. ^#^ *P* < 0.05, ^##^ *P* < 0.01, ^###^ *P* < 0.001 compared with DOX.

Multiple studies have substantiated the antioxidant properties of NBP and its neuroprotective effects. To investigate the potential therapeutic benefit of NBP on DICT, we developed both *in vivo* and *in vitro* models of myocardial injury induced by DOX. The molecular structure of NBP is illustrated in Figure 1A.

First, at the *in vivo* level, we divided the mice into two groups: the control group (CON) and the experimental group, which was further stratified into three subgroups: the DOX-induced model group without NBP intervention, the low-dose (25 mg/kg) NBP treatment group and the high-dose (50 mg/kg) NBP treatment group. The mice in the experimental group received an intraperitoneal injection of DOX (4 mg/kg) once a week for 3 weeks, and those in the administration groups were treated with NBP every other day for 3 weeks (25 mg/kg or 50 mg/kg, respectively, i.p.) after the first injection of DOX.

Prior to euthanasia, transthoracic echocardiography was performed to assess the cardiac function parameters across each group. Quantitative analysis demonstrated that NBP administration significantly ameliorated DOX-induced cardiac dysfunction, as evidenced by marked improvements in key cardiac functional parameters, including the ejection fraction (EF%) and left ventricular shortening fraction (FS%) (Figures 1B–D). Serum biomarkers of myocardial injury, specifically creatine kinase MB isoenzyme (CK-MB) and lactate dehydrogenase (LDH), were quantitatively analyzed, with the experimental results presented in Figures 1E,F. Biochemical analysis revealed a significant attenuation of CK-MB and LDH release in NBP-treated groups compared to the DOX-induced model group. These findings collectively indicate that NBP exerts cardioprotective effects against DOX-induced myocardial injury, potentially through the mitigation of cardiomyocyte damage.

### 3.2 NBP treatment alleviated DOX-induced cardiac fibrosis

**FIGURE 2:**
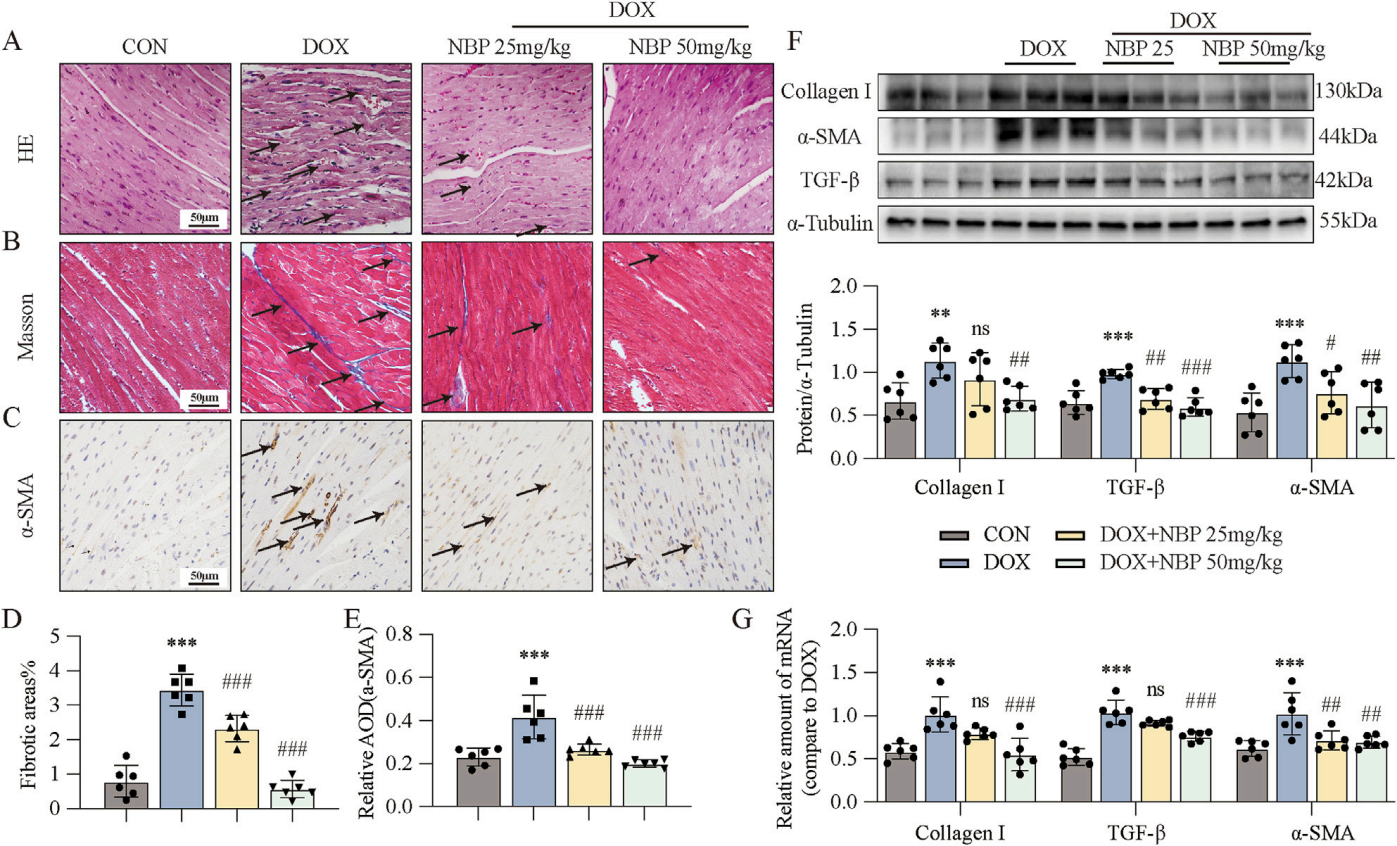
NBP treatment alleviated DOX-induced cardiac fibrosis. **(A)** Representative hematoxylin-eosin (H&E) staining myocardial tissue sections (n = 6 mice per group, 1 section per mouse) were performed to demonstrate the protective effects of NBP against DOX-induced structural alterations, with arrows indicating specific areas of myocardial fiber disorganization and structural deficits. **(B)** Fibrosis in the heart tissues of each group of mice was evaluated through representative micrographs obtained from Masson staining. **(C)** Representative micrographs of anti-α-smooth muscle actin (α-SMA) staining were obtained to visualize the accumulation of α-SMA, with arrows indicating the accumulation areas. **(D,E)** Quantification of fibrotic areas (%) and α-SMA abundance via Masson’s trichrome staining and anti-α-SMA staining (n = 6 mice per group, 1 section per mouse). **(F)** Representative Western blot analysis of collagen type I (Col-I), α-SMA, and transforming growth factor-beta (TGF-β) in heart tissues is shown, with α-Tubulin as the loading control. Bottom, densitometric quantification of the data in **(F)** (n = 6 per group). **(G)** qRT-PCR was used to quantify the mRNA levels of Col-I, α-SMA, and TGF-β1 in cardiac tissues (n = 6 per group). The data are presented as the means ± SDs. Different groups were compared with a one-way analysis of variance (ANOVA). *P < 0.05, **P < 0.01, ***P < 0.001 compared with CON. ^#^ *P* < 0.05, ^##^ *P* < 0.01, ^###^ *P* < 0.001 compared with DOX.

As myocardial injury progresses, significant pathophysiological alterations in cardiac architecture and tissue composition emerge, ultimately resulting in ventricular remodeling and progressive myocardial fibrosis. Histopathological evaluation by hematoxylin-eosin (H&E) staining demonstrated distinct morphological changes in myocardial tissue architecture across each group, revealing characteristic pathological features associated with disease progression (Figure 2A). Histopathological analysis revealed distinct morphological characteristics among each group: the control group maintained normal myocardial, while the DOX-treated group exhibited significant disruption of myocardial fiber organization, characterized by disordered arrangement and fragmentation of cardiac muscle fibers. However, NBP treatment alleviated these pathological alterations. To further investigate the antifibrotic properties of NBP, we performed Masson (Figure 2B) and immunohistochemical staining of α-SMA (Figure 2C). Quantitative analysis using ImageJ software (NIH, USA) demonstrated significant differences in fibrotic area and α-SMA expression levels among groups, as presented in Figures 2D,E. These images revealed that NBP reduced collagen fiber deposition (blue area) and α-SMA accumulation. We subsequently performed Western blotting and PCR to estimate the protein and mRNA levels of fibrotic genes, including α-smooth muscle actin (α-SMA), transforming growth factor-beta (TGF-β), and collagen type I (Col-I), both *in vivo* and *in vitro*. At the cell level, we exposed H9C2 cells to 1 μM DOX to simulate the onset of DICT. On the basis of the results of the previous experiments in our lab, we selected 20 μM and 40 μM concentrations for subsequent experiments. Western blot analysis revealed a significant upregulation of fibrotic markers, including α-SMA, TGF-β, and Col-I, in response to DOX-induced cardiac injury. However, pharmacological intervention with NBP effectively attenuated this protein overexpression (Figure 2F; Supplementary Figure S1A). In parallel with the WB results, qRT-PCR analysis revealed that NBP treatment significantly downregulated the transcriptional levels of these fibrotic markers compared to the DOX group (Figure 2G; Supplementary Figure S1B). These findings indicate that NBP exerts potent antifibrotic effects through the modulation of key fibrotic pathways in DOX-induced cardiomyopathy.

### 3.3 Effects of NBP on inflammation and apoptosis in DOX-induced cardiotoxicity

**FIGURE 3:**
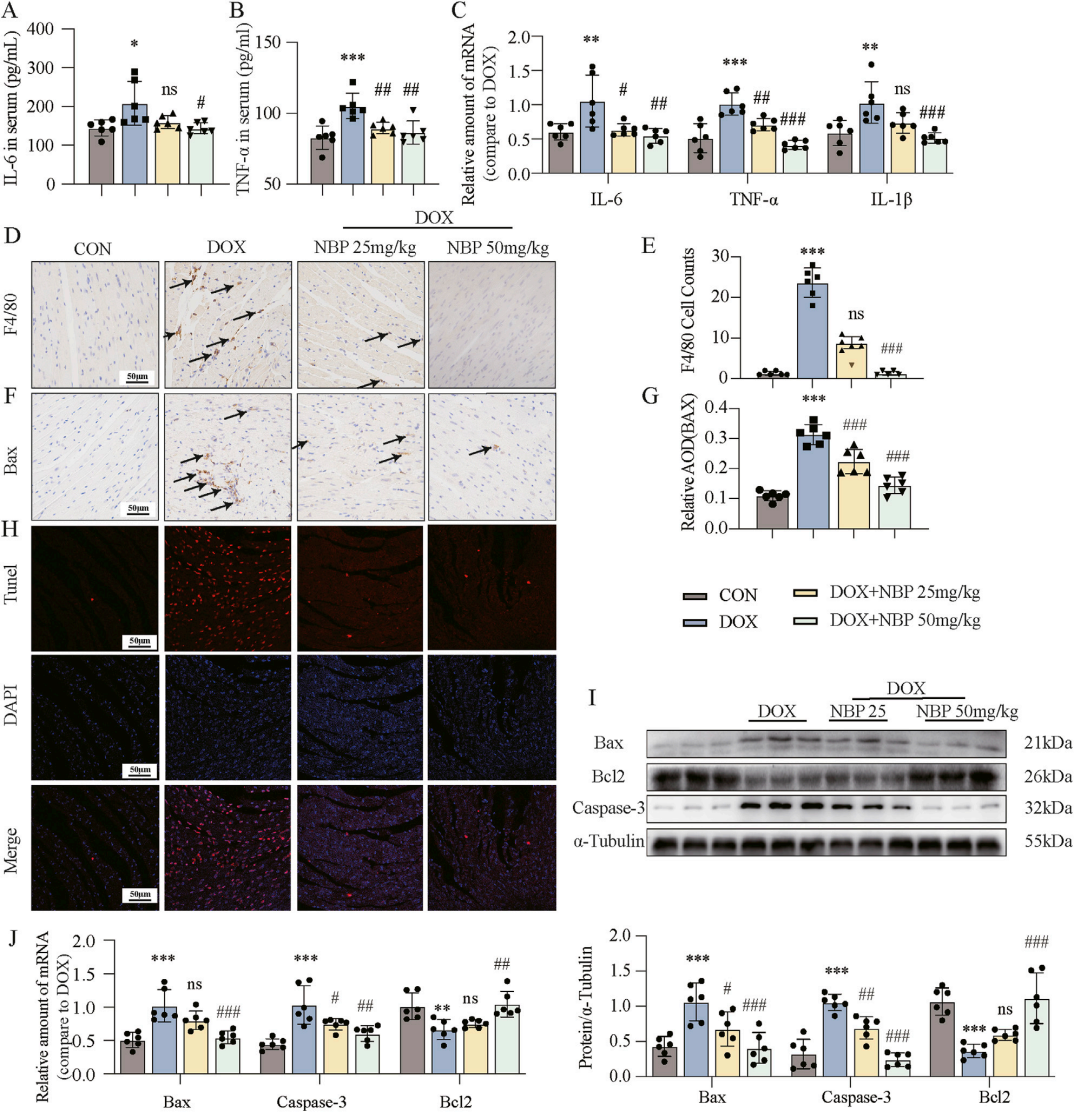
NBP treatment alleviated DOX-induced cardiac inflammatory and apoptotic responses. **(A,B)** Detection of interleukin-6 (IL-6) and tumor necrosis factor-alpha (TNF-α) in the serum through enzyme-linked immunosorbent assay (ELISA) kits (n = 6 per group). Different groups were compared with a one-way analysis of variance (ANOVA). **(C)** qRT-PCR was conducted to measure the mRNA levels of IL-6, TNF-α, and interleukin-1 beta (IL-1β) in cardiac tissues (n = 6 per group). Different groups were compared with a one-way analysis of variance (ANOVA). **(D)** Representative micrographs of anti-F4/80-stained samples were obtained to visualize the infiltration of F4/80, with arrows indicating the infiltration areas. **(E)** Quantitative analysis of the levels of F4/80 (n = 6 mice per group, 1 section per mouse). Different groups were compared with Kruskal-Wallis test. **(F)** Representative micrographs illustrating anti- Bcl-2-associated X protein (Bax) staining, with arrows indicating the accumulation of Bax. **(G)** Quantitative analysis of Bax (n = 6 mice per group, 1 section per mouse). Different groups were compared with a one-way analysis of variance (ANOVA). **(H)** Representative micrographs displaying terminal deoxynucleotidyl transferase dUTP nick end labeling (TUNEL) staining. **(I)** Western blot analysis revealed the expression of the key proteins Bax, B-cell lymphoma/leukemia 2 (Bcl2), and cysteine-aspartic acid protease 3 (Caspase-3) in cardiac tissues, with α-Tubulin used as the loading control. Bottom, densitometric quantification of the data in **(I)** (n = 6 per group). Different groups were compared with a one-way analysis of variance (ANOVA). **(J)** qRT-PCR was conducted to measure the mRNA levels of Bax, Bcl2, and Caspase-3 in cardiac tissues (n = 6 per group). Different groups were compared with a one-way analysis of variance (ANOVA). The data are presented as the means ± SDs. *P < 0.05, **P < 0.01, ***P < 0.001 compared with CON. ^#^ *P* < 0.05, ^##^ *P* < 0.01, ^###^ *P* < 0.001 compared with DOX.

Inflammation and apoptosis represent pivotal pathological hallmarks of DOX-induced cardiotoxicity. To elucidate the anti-inflammatory properties of NBP, we investigated the expression levels of key pro-inflammatory cytokines, including interleukin-6 (IL-6), tumor necrosis factor-alpha (TNF-α), and interleukin-1 beta (IL-1β), which are known to play pivotal roles in inflammatory responses. Subsequently, quantitative assessment of serum IL-6 and TNF-α levels across all experimental groups was performed via enzyme-linked immunosorbent assay (ELISA) kits. The levels of these substances were markedly elevated in the DOX group, but they exhibited a decline after NBP treatment (Figures 3A,B). Moreover, NBP treatment significantly attenuated the DOX-induced mRNA upregulation of IL-6, TNF-α, and IL-1β in both heart tissues and H9C2 cells (Figure 3C; Supplementary Figure S2A). Immunohistochemical staining for F4/80, a well-established macrophage marker, was then performed (Figure 3D). The results showed that NBP treatment attenuated the DOX-induced accumulation of F4/80-positive cells, and the statistical analysis of positive staining is depicted in Figure 3E.

To further investigate the anti-apoptotic effects of NBP, we performed immunohistochemical staining and TUNEL assay at the animal level. The results demonstrated that NBP treatment reduced the DOX-induced accumulation of Bax-positive cells (Figures 3F,G) and inhibited DOX-induced cardiomyocyte apoptosis (Figure 3H). Additionally, Western blotting and PCR were carried out to examine the protein and mRNA levels of the antiapoptotic gene B-cell lymphoma/leukemia 2 (Bcl2) and the proapoptotic genes Bcl-2-associated X protein (Bax) and cysteine-aspartic acid protease 3 (Caspase-3) both *in vivo* and *in vitro*. Western blot analysis revealed that NBP administration reversed the decrease in Bcl2 protein levels and the increase in Bax and Caspase-3 expression induced by DOX treatment (Figure 3I; Supplementary Figure S2B). Similarly, positive effects of NBP on the mRNA expression of these genes were also observed by PCR analysis (Figure 3J; Supplementary Figure S2C). These findings collectively indicate that NBP treatment alleviates the inflammatory and apoptotic responses induced by DOX in the heart.

### 3.4 NBP treatment alleviated DOX-induced cardiac oxidative stress and mitochondrial damage

**FIGURE 4:**
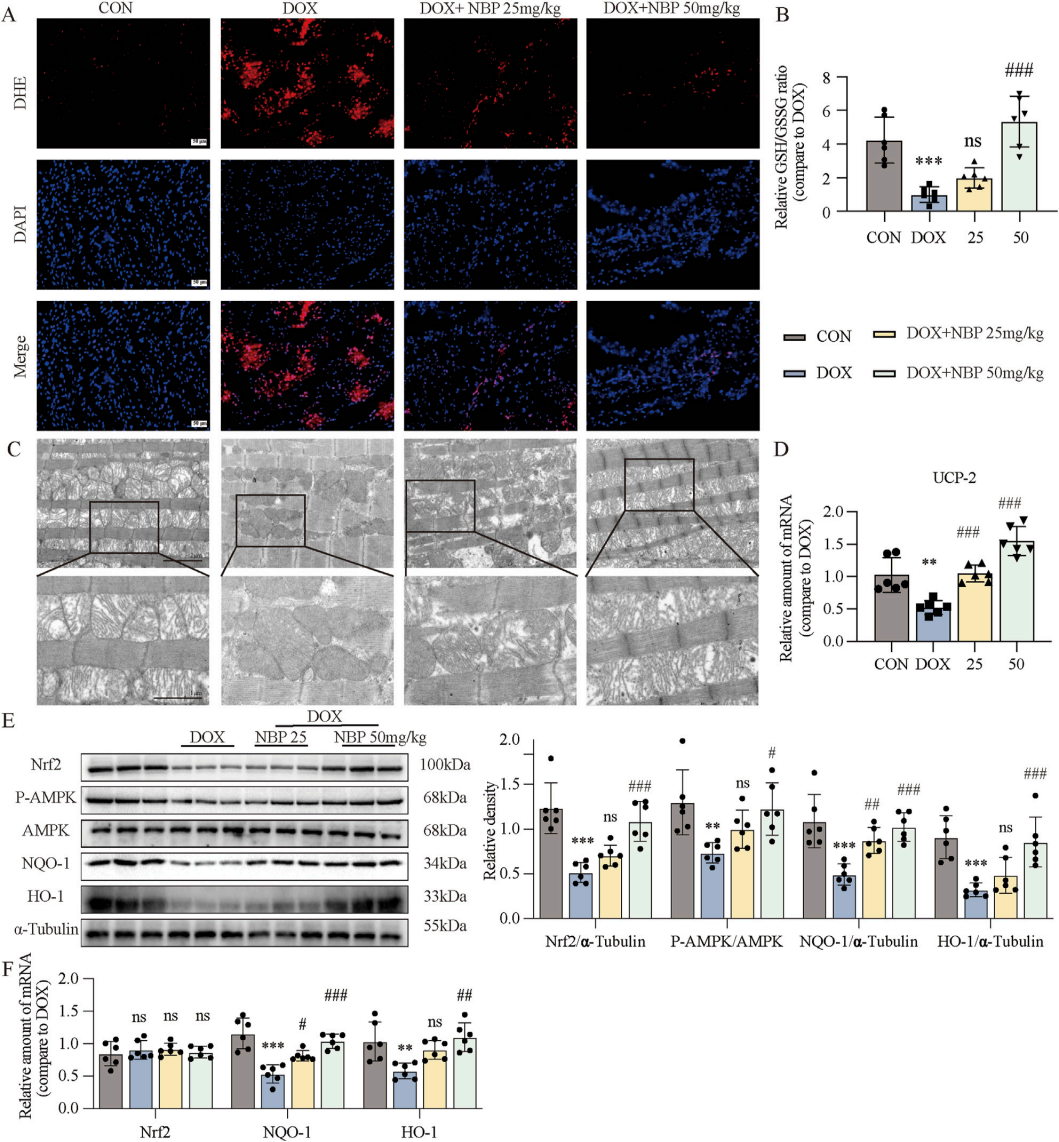
NBP treatment alleviated DOX-induced cardiac oxidative stress (OS) and mitochondrial damage. **(A)** Representative dihydroethidium (DHE) staining images of cardiac tissues (n = 6 mice per group, 1 section per mouse). **(B)** Measurment of the glutathione (GSH)/oxidized glutathione (GSSG) ratio in the cardiac tissues through GSH and GSSG assay kit (n = 6 per group). **(C)** Representative transmission electron microscopy (TEM) images of cardiac tissue mitochondria (n = 3 mice per group, 2 section per mouse). **(D)** qRT-PCR was carried out to measure uncoupling protein 2 (UCP-2) mRNA level in cardiac tissues (n = 6 per group). **(E)** Western blot analysis of nuclear factor erythroid 2-related factor 2 (Nrf2), phosphorylated AMP-activated protein kinase (P-AMPK), NAD(P)H quinone dehydrogenase 1 (NQO-1), and Heme oxygenase-1 (HO-1) proteins in cardiac tissues was conducted, with AMP-activated protein kinase (AMPK) and α-Tubulin used as loading controls. Right, densitometric quantification of the data in **(E)** (n = 6 per group). **(F)** qRT-PCR was conducted to measure Nrf2, NQO-1 and HO-1 mRNA levels in cardiac tissues (n = 6 per group). The data are presented as the means ± SDs. Different groups were compared with a one-way analysis of variance (ANOVA). *P < 0.05, **P < 0.01, ***P < 0.001 compared with CON. ^#^ *P* < 0.05, ^##^ *P* < 0.01, ^###^ *P* < 0.001 compared with DOX.

Oxidative stress (OS) is the term used to describe an imbalance between oxidation and antioxidation in the body. The continuous accumulation of oxidative substances such as ROS leads to damage and disease. To determine whether NBP could alleviate OS caused by DOX, we performed DHE staining and glutathione (GSH)/oxidized glutathione (GSSG) analysis of heart tissues. DHE was used to label superoxide anions in heart tissues and H9C2 cells from each group (Figure 4A; Supplementary Figure S3A). The intensity of red fluorescence in cells treated with only DOX increased significantly, indicating a high level of OS. In contrast, NBP administration significantly reduced the fluorescence intensity. And NBP administration also deficiency attenuated DOX-induced downregulation of GSH/GSSG ratio (Figure 4B). These results confirmed that NBP could mitigate DOX-induced OS.

Mitochondrial dysfunction, a direct consequence of OS, further exacerbates the imbalance in cellular homeostasis. By TEM, we observed significant ultrastructural alterations in cardiac mitochondria from the DOX-treated group, characterized by shortened, reduced, or absent. In contrast, mitochondrial morphology in NBP-treated groups exhibited preserved structural integrity, with relatively well-organized and intact cristae (Figure 4C). We also utilized JC-1 staining on H9C2 cells to assess the potential of the mitochondrial membrane (Supplementary Figure S3B). The images revealed that incubation with DOX led to a significant increase in the intensity of green fluorescence and a decrease in red fluorescence intensity, whereas NBP treatment reversed these changes. We subsequently performed qRT-PCR assays to analyze the mRNA expression of uncoupling protein 2 (UCP-2) both *in vivo* and *in vitro.* UCP-2, a member of the mitochondrial uncoupling protein family, is known to regulate mitochondrial function by reducing ROS production. The results indicated that NBP therapy reversed the decrease in UCP-2 mRNA expression induced by DOX (Figure 4D; Supplementary Figure S3C). Additionally, we assessed the ATP content in H9C2 cells from each group to evaluate mitochondrial function. As shown in Supplementary Figure S3D, NBP improved the ability of mitochondria to produce ATP after exposure to DOX. These results confirmed that NBP can preserve the standard structure and function of mitochondria.

We further analyzed the protein and mRNA levels of Nuclear factor erythroid 2-related factor 2 (Nrf2), Heme oxygenase-1 (HO-1), NAD(P)H quinone dehydrogenase 1 (NQO-1), and the phosphorylation level of AMP-activated protein kinase (AMPK) in heart tissue and H9C2 cells, to assess the antioxidant ability of NBP. Western blot assays demonstrated that NBP reversed the decrease in the protein or phosphorylation levels of these antioxidant genes caused by DOX (Figure 4E; Supplementary Figure S3E). Interestingly, there was no significant alterations in the mRNA level of Nrf2 across the different groups. In contrast, NBP treatment significantly upregulated the mRNA levels of HO-1 and NQO-1, compared to DOX-treated group (Figure 4F; Supplementary Figure S4F). On the basis of these results, we assumed that regulating the posttranslational modification of Nrf2 may be a potential molecular mechanism of NBP.

### 3.5 Inhibition of Keap1/Nrf2 binding by NBP: effects on ubiquitination and protein levels

The level of Nrf2 are predominantly regulated by Keap1, which functions as a key repressor of Nrf2 activity (Dinkova-Kostova et al., 2002). Targeting the Keap1/Nrf2 signaling pathway has emerged as a promising therapeutic strategy for the treatment of various diseases (Crisman et al., 2023). When Nrf2 is bound to Keap1 in the cytoplasm, it undergoes ubiquitination and subsequent degradation by the proteasome. Consequently, it cannot translocate into the nucleus to activate the transcription of its downstream antioxidant genes, such as NQO-1 and HO-1. On the basis of this degradation pathway of Nrf2, we hypothesized that NBP may inhibit the binding of Keap1 and Nrf2 to exert its anti-OS, anti-inflammatory, and antiapoptotic effects.

**FIGURE 5:**
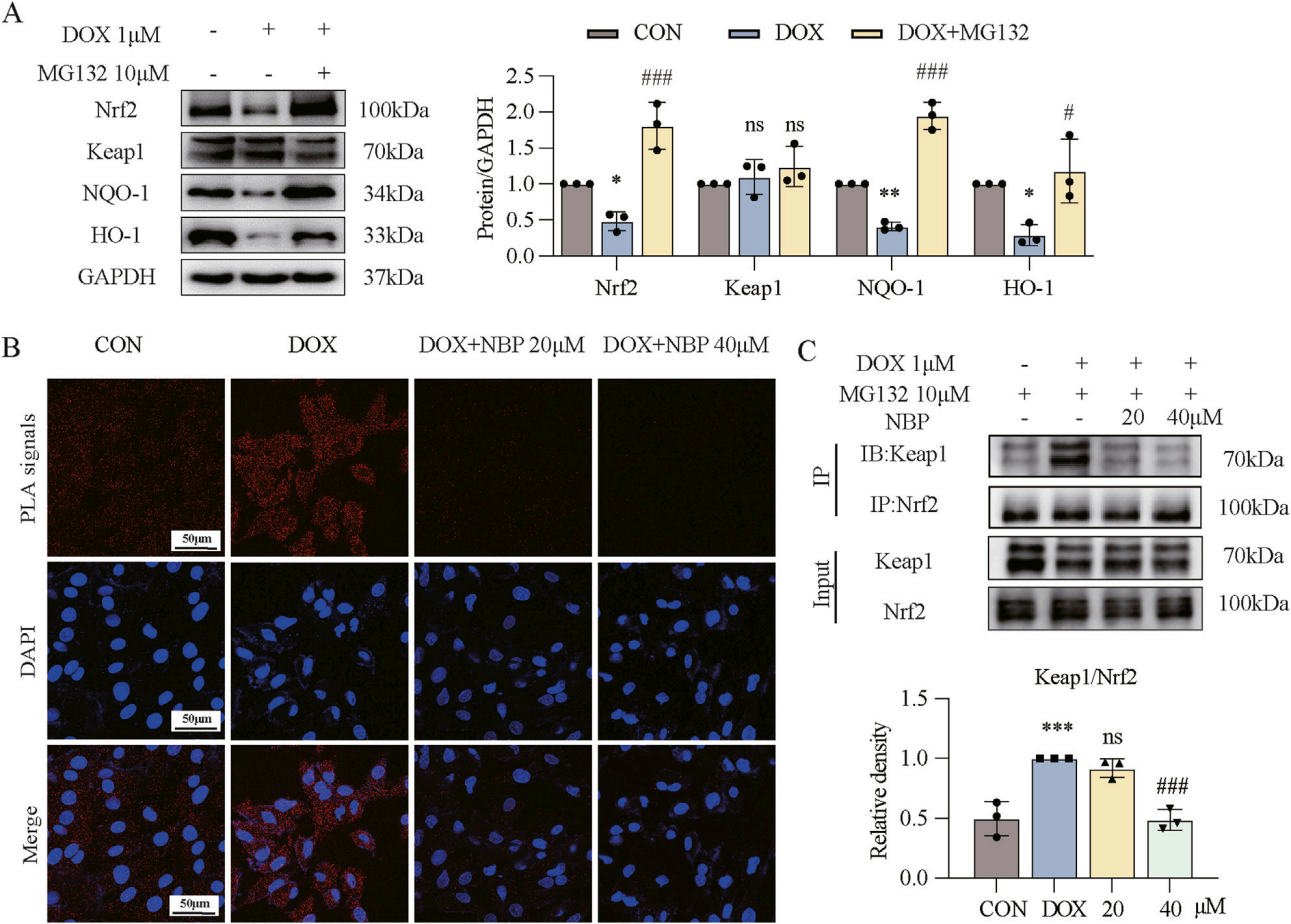
NBP inhibited Nrf2/Keap1 binding to alleviate DOX-induced myocardial injury. **(A)** Western blot analysis of the protein levels of antioxidant genes (Nrf2, Keap1, NQO-1, and HO-1) in H9C2 cells. H9C2 cells underwent a 1-hour pretreatment with MG132, followed by a 24-hour stimulation with DOX (1 μM). Right, densitometric quantification for **(A)** (n = 3). **(B)** Effect of NBP on Nrf2/Keap1 binding in H9C2 cells evaluated through proximity ligation assay (PLA) immunofluorescence. H9C2 cells underwent a 1-hour pretreatment with NBP, followed by a 12-hour stimulation with DOX (1 μM). Representative immunofluorescence images from three independent experiments are shown. The PLA signals are red, while the DAPI nuclear stain is blue. **(C)** For Co-immunoprecipitation (Co-IP), H9C2 cells underwent a 1-hour pretreatment with NBP, followed by a 12-hour stimulation with DOX (1 μM). Bottom, densitometric quantification for **(C)** (n = 3). The data are presented as the means ± SDs. Different groups were compared with a one-way analysis of variance (ANOVA). *P < 0.05, **P < 0.01, ***P < 0.001 compared with CON. ^#^ *P* < 0.05, ^##^ *P* < 0.01, ^###^ *P* < 0.001 compared with DOX.

To investigate the potential occurrence of ubiquitination-mediated protein degradation in our cellular system, we coincubated the H9C2 cells with DOX and proteasomal inhibition MG132. The Western blot analysis revealed that MG132 treatment effectively abolished the DOX-induced reduction in protein expression levels of Nrf2 and its downstream targets NQO-1 and HO-1 (Figure 5A), which provides compelling evidence that DOX-induced Nrf2 downregulation is mediated through ubiquitin-proteasome system activation. To investigate the potential inhibitory effect of NBP on the Keap1/Nrf2 interaction, we then conducted a PLA assay. As shown in Figure 5B, the red fluorescence enhancement induced by DOX was significantly reduced after NBP treatment. To further validate this finding, we performed Co-IP assays *in vitro* (Figure 5C). The results demonstrated that NBP treatment significantly reduced the binding affinity between Keap1 and Nrf2 compared to the DOX-treated group. Collectively, these results suggest that NBP disrupts the Keap1/Nrf2 interaction, providing a potential mechanism for its antioxidant effects.

### 3.6 Cardioprotective effects of NBP depend on the Nrf2 signaling pathway

**FIGURE 6:**
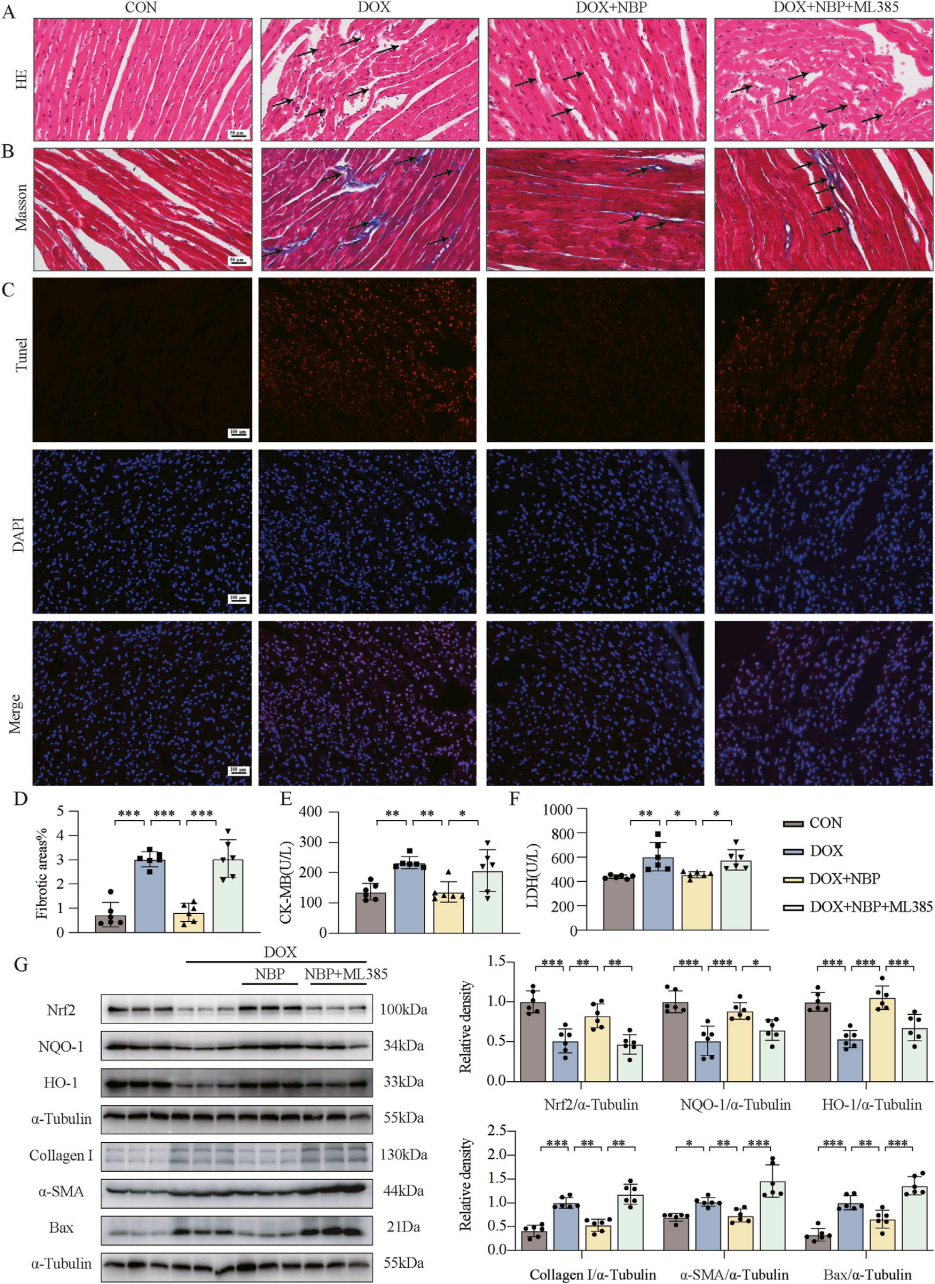
The cardioprotective effect of NBP depends on the Nrf2 signaling pathway. **(A)** Representative hematoxylin-eosin (H&E) staining myocardial tissue sections (n = 6 mice per group, 1 section per mouse) were performed to demonstrate the potential mechanism of NBP against DOX-induced structural alterations, with arrows indicating specific areas of myocardial fiber disorganization and structural deficits. **(B)** Fibrosis in the heart tissues of each group of mice was evaluated through representative micrographs obtained from Masson staining. **(C)** Representative micrographs displaying terminal deoxynucleotidyl transferase dUTP nick end labeling (TUNEL) staining. **(D)** Quantification of fibrotic areas (%) via Masson’s trichrome staining (n = 6 mice per group, 1 section per mouse). **(E,F)** The concentrations of creatine kinase-isoenzyme (CK-MB) and lactate dehydrogenase (LDH) in murine serum samples were determined using dedicated kits (n = 6 per group). **(G)** Western blot analysis was utilized to assess the protein levels of Nrf2, NQO-1, HO-1, Collagen I, α-SMA, and Bax in heart tissues. α-Tubulin was used as a loading control (n = 6). The data are presented as the means ± SDs. Different groups were compared with a one-way analysis of variance (ANOVA). *P < 0.05, **P < 0.01, ***P < 0.001.

To further validate the involvement of the Nrf2 signaling pathway in mediating the pharmacological effects of NBP, we utilized ML385, a specific inhibitor of Nrf2 transcriptional activity. First, at the *in vivo* level, we divided the mice into two groups: the control group (CON) and the experimental group, which was further stratified into three subgroup: the DOX-induced model group without NBP intervention, the 50 mg/kg NBP treatment group and combined therapy of 50 mg/kg NBP and 30 mg/kg ML385 treatment group. The mice in the experimental group received an intraperitoneal injection of DOX (4 mg/kg) once a week for 3 weeks. H&E staining revealed significant alterations in myocardial tissue morphology among experimental groups across each group (Figure 6A). To further investigate the potential therapeutic mechanism of NBP, we performed Masson staining (Figures 6B,D) and TUNEL assay (Figure 6C). CK-MB and LDH in serum were quantitatively analyzed, with the experimental results presented in Figures 6E,F. These results collectively demonstrate that the therapeutic effects of NBP on DOX-induced myocardial injury were effectively abolished by ML385 co-administration.

Western blot analysis revealed both *in vivo* and *in vitro* that co-administration with ML385 significantly attenuated the effects of NBP-induced upregulation of antioxidant proteins (Nrf-2, NQO-1, HO-1). Moreover, the protein levels of fibrotic markers (α-SMA, collagen-I) and the pro-apoptotic protein Bax were significantly elevated in the ML385 + NBP co-treatment group compared to the group treated with NBP alone (Figure 6G; Supplementary Figure S4A). These findings suggest that when the expression of Nrf2 and its downstream genes was inhibited, NBP could not relieve the OS, fibrosis, or apoptosis of cardiomyocytes induced by DOX. DHE staining of H9C2 cells further demonstrated that a significant increase in ROS levels in the ML385 + NBP co-treatment group compared to the group treated with NBP alone (Supplementary Figure S4B). Additionally, as shown in Supplementary Figure S4C, co-incubation with NBP and ML385 led to weakened red fluorescence intensity and increased green fluorescence intensity in cells, as determined by JC-1 staining. These findings indicate that the ability of NBP to improve mitochondrial function disappeared. Collectively, these findings underscore the critical role of the Nrf2 pathway in mediating the cardioprotective effects of NBP.

## 4 Discussion

With the progression in cancer therapeutics, patient survival rates have significantly improved; however, this advancement has concurrently led to an increase in morbidity and mortality associated with treatment- related adverse effects (Ferlay et al., 2013; Miller et al., 2022). Notably, CVDs have emerged as the predominant side effects, raising substantial concerns due to their potential to precipitate premature morbidity and mortality among cancer survivors (Ewer and Ewer, 2015). DOX, an anthracycline antibiotic, is recognized for its broad-spectrum antitumor efficacy (Lee et al., 2014) and is used as a first-line chemotherapy drug in the clinical treatment of various types of cancer, including hematologic and solid cancer. Despite its high therapeutic index in oncological treatment, the cardiotoxic effects of DOX are significant and predominantly manifest as heart failure and cardiac arrhythmias (Boddicker et al., 2021). Clinical studies have shown that DOX-induced cardiotoxicity is concentration-related. For instance, the incidence of congestive heart failure is reported at a mere 5% with a cumulative lifetime DOX dose of 400 mg/m^2^. This risk escalates exponentially with increasing cumulative doses, reaching 26% at 550 mg/m^2^ and 48% at 700 mg/m^2^ (Swain et al., 2003). In this study, transthoracic echocardiography revealed a significant decline in cardiac function in DOX-treated murine models, accompanied by a marked elevation in serum biochemical markers (CK-MB and LDH), indicative of myocardial cell injury and necrosis.

Extensive research has elucidated the molecular mechanisms underlying DOX-induced myocardial injury, primarily involving various stress responses that culminate in ROS generation, mitochondrial dysfunction, and apoptosis (Gu et al., 2018; Zilinyi et al., 2018). ROS-mediated oxidative stress is a critical contributor to myocardial injury, causing damage to DNA, RNA, lipids and proteins, ultimately triggering cardiomyocyte apoptosis (Wang et al., 2023). Our findings demonstrated that the DOX-treated mice and cardiomyocytes exhibited sustained oxidative stress, accompanied by elevated inflammation, apoptosis, and mitochondrial dysfunction. Given the pivotal role of ROS in DOX-induced cardiotoxicity, we propose that antioxidant agents hold significant therapeutic potential for mitigating this condition.

The antioxidant activities of NBP have been reported in multiple studies (Liao et al., 2018; Wang et al., 2016). Considering that NBP is a candidate drug for DOX-induced cardiotoxicity, we demonstrated the cardioprotective effect of NBP both *in vivo* and *in vitro*. *In vivo*, NBP was administered intraperitoneally (25 mg/kg, 50 mg/kg) to DOX-treated mice, while *in vitro,* DOX-treated cells were coincubated with NBP (20 μM, 40 μM). Our results demonstrated that NBP administration ameliorated DOX-induced pharmacological alterations, including cardiac and mitochondrial dysfunction, as well as inflammation, apoptosis, fibrosis, and oxidative stress both *in vivo* and *in vitro*. Notably, although Nrf2 mRNA expression remained unchanged in heart tissue or cardiomyocytes, Nrf2 protein level was significantly elevated in the NBP-treated group. On the basis of this phenomenon, we hypothesized that the cardioprotective effect of NBP might be related to the posttranslational modification of Nrf2.

The Nrf2/ARE pathway plays a pivotal role in maintaining cellular homeostasis by regulating ROS-induced stress, thereby mitigating oxidative stress-related diseases, including CVD (Cheng et al., 2020). Nrf2, a transcription factor comprising 605 amino acids, is essential for redox-sensitive processes and contains seven conserved Nrf2-ECH homology (Neh) domains which are critical for its function (Moi et al., 1994). Keap1, acting as a Cul3-dependent E3 ubiquitin ligase adaptor, regulates Nrf2 ubiquitination due to its cysteine-rich structure (Cuadrado et al., 2019). Under physiological conditions, Nrf2 signaling is tightly regulated. The Nrf2 protein binds to Keap1 via the Neh2 domain, forming an inactive cytoplasmic complex that undergoes proteasomal degradation upon ubiquitination by the CUL3-Keap1 complex (Suzuki et al., 2019). Conversely, the functional conformation of Keap1 is altered, following which Nrf2 dissociates from the Keap1 complex in the cytosol (Sengoku et al., 2022). Nrf2 then translocates into the nucleus and binds to small Maf proteins and AREs via the Neh1 domain (Katsuoka and Yamamoto, 2016) to activate the expression of downstream target genes (such as HO-1 and NQO-1) (Guo and Mo, 2020). Therefore, the balance of oxidation and antioxidants maintains the body in a normal state. However, persistent OS disrupts this equilibrium, leading to Nrf2 sequestration and impaired antioxidant function, contributing to DOX-induced myocardial injury (Shrestha et al., 2024).

Considering the regulatory mechanism of Nrf2, we hypothesized that NBP may exert its pharmacological activity by inhibiting the binding of Keap1 and Nrf2. Therefore, we conducted co-immunoprecipitation and PLA assays to investigate the effect of NBP on the binding of Nrf2 and Keap1, as shown in Figure 5. We found that the binding of these two proteins was significantly inhibited by NBP administration. Our study also demonstrated the crucial role of the Nrf2 signaling pathway in the pharmacological activity of NBP via ML385, a compound that specifically inhibits the expression of Nrf2 and its downstream genes, as shown in Figure 6.

In conclusion, our studies, conducted both *in vivo* and *in vitro*, demonstrated that NBP could alleviate DOX-induced inflammation, fibrosis, apoptosis, and mitochondrial injury by regulating the oxidative stress state. Furthermore, the specific molecular mechanism of NBP was explored and verified. Co-immunoprecipitation and PLA revealed that the pharmacological activity of NBP could be attributed to its ability to inhibit Keap1/Nrf2 binding. These findings highlight the potential therapeutic value of NBP as a promising treatment for DOX-induced cardiotoxicity. It also provides essential insights into the molecular basis underlying the beneficial effects of NBP and suggest that Nrf2/Keap1 may be a potential therapeutic target for DOX-induced cardiac complications. However, this study has limitations, including the need to evaluate the long-term effects and potential dependence associated with NBP treatment, which warrant further investigation for managing this condition.

## Data Availability

The original contributions presented in the study are included in the article/Supplementary Material, further inquiries can be directed to the corresponding authors.
