# Supplementary material for: Dl-3-n-butylphthalide attenuates DOX-induced cardiotoxicity in mice by inhibiting Nrf2/Keap1 complex formation
Source: Front Pharmacol. 2025 Apr 29;16:1542296. doi: 10.3389/fphar.2025.1542296 (PMC12069325; doi:10.3389/fphar.2025.1542296)
Supplement: Supplementary file 1 [file DataSheet1.docx]

**Supplementary Material**


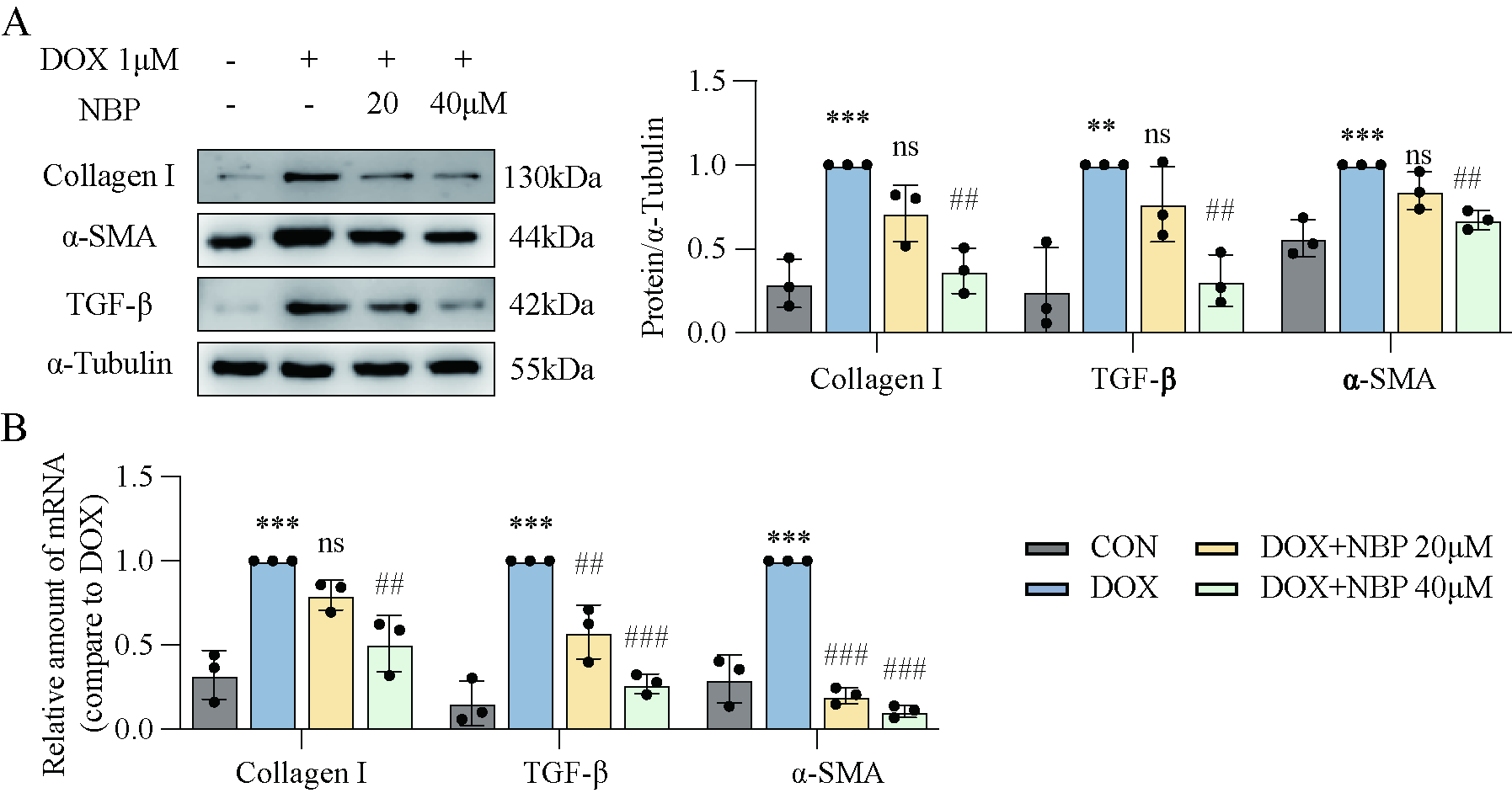


**Fig. S1. NBP treatment alleviated DOX-induced cardiac fibrosis.** (A) H9C2 cells underwent a 1-hour pretreatment with NBP, followed by a 24-hour stimulation with DOX (1 μM). A representative Western blot analysis of collagen type I (Col-I), α-SMA, and transforming growth factor-beta (TGF-β) in cells is shown, with α-Tubulin as the loading control. **Right**, densitometric quantification of the data in (A). (B) H9C2 cells underwent a 1-hour pretreatment with NBP, followed by a 24-hour stimulation with DOX (1 μM). A qRT-PCR assay was carried out to measure the mRNA levels of Col-I, TGF-β, and α-SMA in H9C2 cells. The data are presented as the means±SDs (n=3). Different groups were compared with a one-way analysis of variance (ANOVA). **P* < 0.05, ***P* < 0.01, ****P* < 0.001 compared with CON. #*P* < 0.05, ##*P* < 0.01, ###*P* < 0.001 compared with DOX.

**
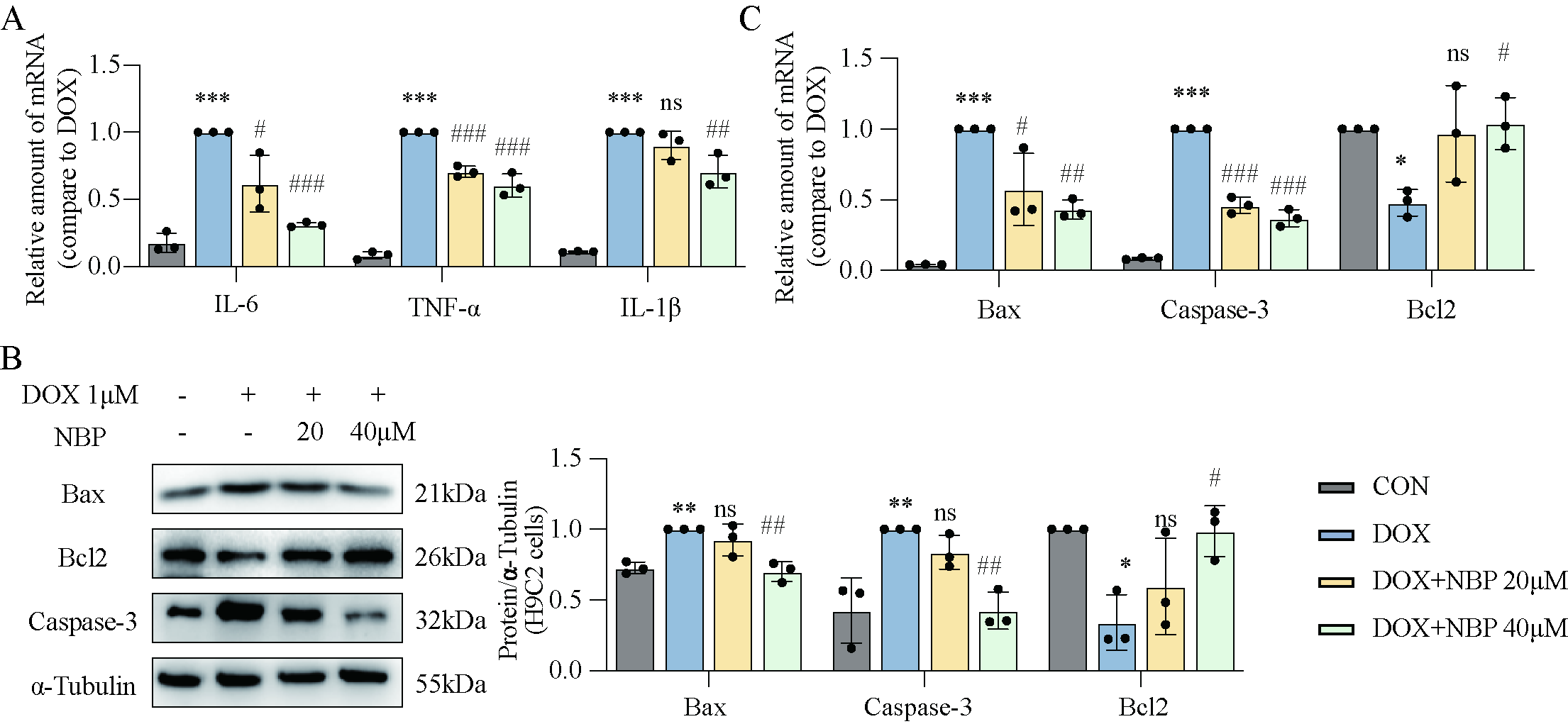
**

**Fig. S2. NBP treatment alleviated DOX-induced cardiac inflammatory and apoptotic responses.** (A) H9C2 cells underwent a 1-hour pretreatment with NBP, followed by a 6-hour stimulation with DOX (1 μM). A qRT-PCR assay was carried out to measure the mRNA levels of interleukin-6 (IL-6), tumor necrosis factor-alpha (TNF-α), and interleukin-1 beta (IL-1β) in H9C2 cells. (B) H9C2 cells underwent a 1-hour pretreatment with NBP, followed by a 12-hour stimulation with DOX (1 μM). A representative Western blot analysis of Bcl-2-associated X protein (Bax), B-cell lymphoma/leukemia 2 (Bcl2), and cysteine-aspartic acid protease 3 (Caspase-3) in H9C2 cells is shown, with α-Tubulin used as the loading control. **Right**, densitometric quantification of the data in (B). (C) H9C2 cells underwent a 1-hour pretreatment with NBP, followed by a 6-hour stimulation with DOX (1 μM). A qRT-PCR assay was carried out to measure the mRNA levels of Bax, Bcl2, and Caspase-3 in H9C2 cells. The data are presented as the means±SDs (n=3). Different groups were compared with a one-way analysis of variance (ANOVA). **P* < 0.05, ***P* < 0.01, ****P* < 0.001 compared with CON. #*P* < 0.05, ##*P* < 0.01, ###*P* < 0.001 compared with DOX.


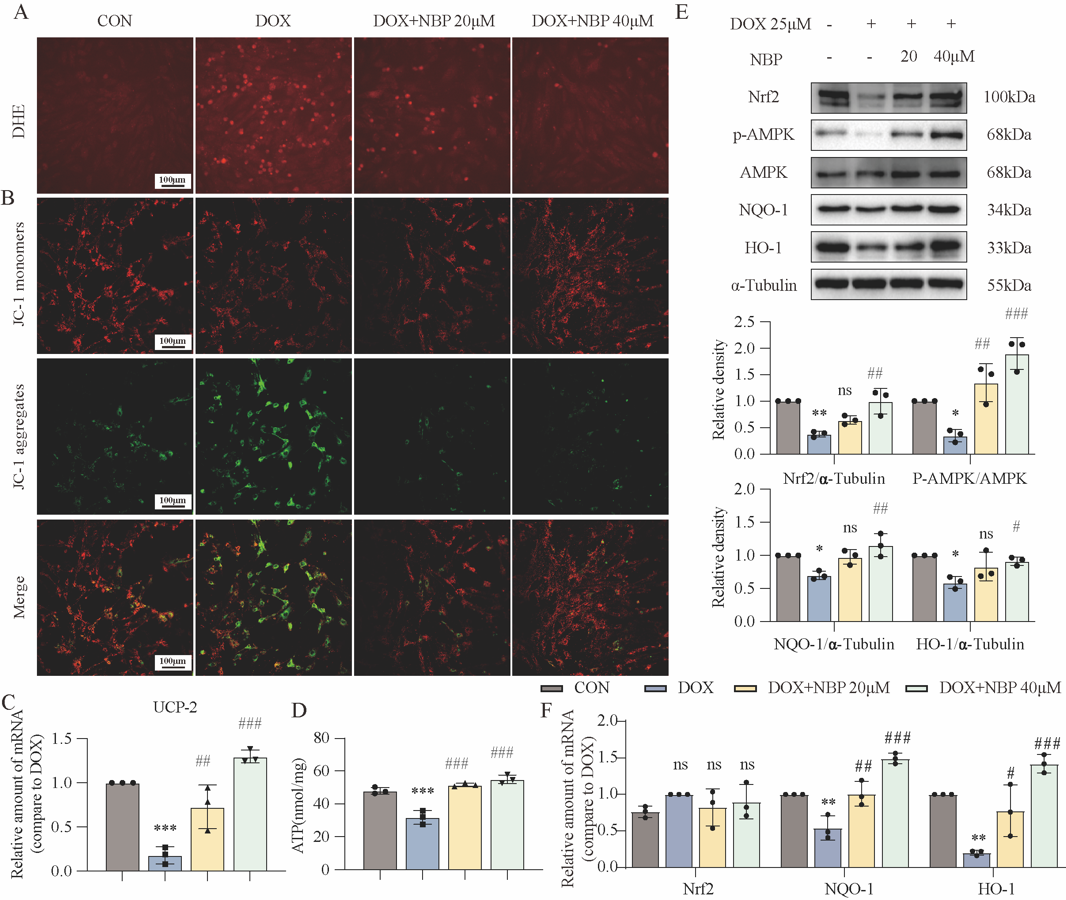


**Fig. S3. NBP treatment alleviated DOX-induced cardiac OS and mitochondrial damage.** (A) Representative images of dihydroethidium (DHE) staining showing the effect of NBP pretreatment for 1 hour followed by stimulation with 1 μM DOX for 12 hours in H9C2 cells. (B) Representative images of JC-1 staining are displayed. (C) H9C2 cells underwent a 1-hour pretreatment with NBP, followed by a 12-hour stimulation with DOX (1 μM). A qRT-PCR assay was performed to measure the mRNA levels of uncoupling protein 2 (UCP-2) in H9C2 cells. (D) The effects of DOX and NBP on the ATP content in H9C2 cells were assessed via an ATP detection kit. (E) H9C2 cells underwent a 1-hour pretreatment with NBP, followed by a 24-hour stimulation with DOX (1 μM). A representative Western blot analysis was used to assess the expression of nuclear factor erythroid 2-related factor 2 (Nrf2), phosphorylated AMP-activated protein kinase (P-AMPK), NAD(P)H quinone dehydrogenase 1 (NQO-1), and Heme oxygenase-1 (HO-1) in H9C2 cells. α-Tubulin and AMP-activated protein kinase (AMPK) served as loading controls. **Bottom**, densitometric quantification of the data in (E). (F) H9C2 cells underwent a 1-hour pretreatment with NBP, followed by a 12-hour stimulation with DOX (1 μM). A qRT-PCR assay was carried out to measure the mRNA levels of Nrf2, NQO-1 and HO-1 in H9C2 cells. The data are presented as the means±SDs (n=3). Different groups were compared with a one-way analysis of variance (ANOVA).**P* < 0.05, ***P* < 0.01, ****P* < 0.001 compared with CON. #*P* < 0.05, ##*P* < 0.01, ###*P* < 0.001 compared with DOX.


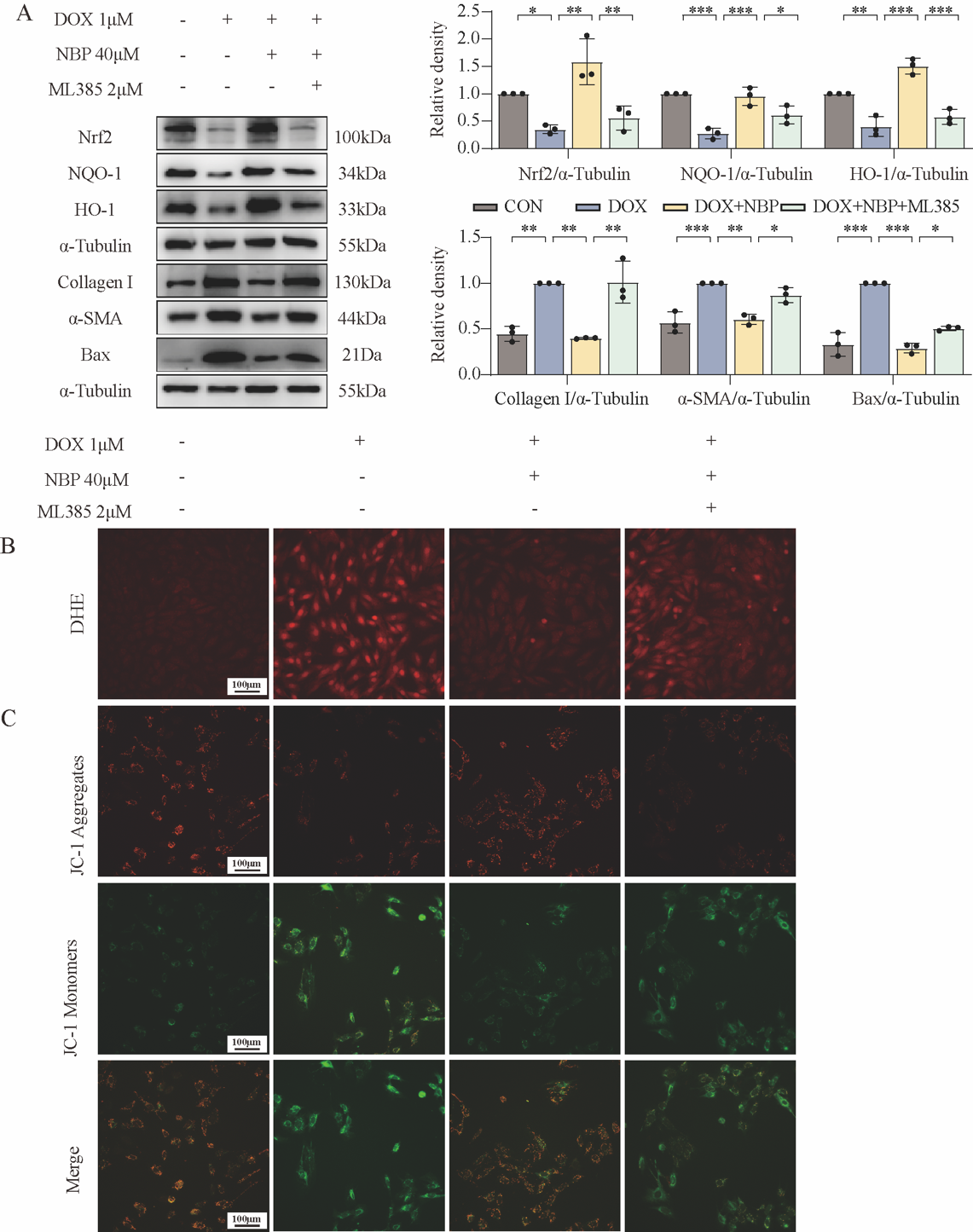


**Fig. S4.** **The cardioprotective effect of NBP depends on the Nrf2 signaling pathway.** To investigate the role of the Nrf2 signaling pathway in NBP treatment in DOX-induced cardiomyopathy, H9C2 cells were pretreated with ML385 (2 μM) for 30 min, followed by NBP treatment for 1 hour and stimulation with DOX (1 μM) for 24 hours (western blot) or 12 hours (DHE and JC-1 staining). (A) Western blot analysis was utilized to assess the protein levels of Nrf2, NQO-1, HO-1, Collagen I, α-SMA, and Bax in H9C2 cells. α-Tubulin was used as a loading control (n=3). Representative micrographs of DHE staining (B) and JC-1 staining (C) from three independent experiments are shown. The data are presented as the means±SDs. Different groups were compared with a one-way analysis of variance (ANOVA). **P* < 0.05, ***P* < 0.01, ****P* < 0.001.

**Table S1.** Primer sequences of the genes in this study.

| Gene | Species | Forward primers | Reverse primes |  |
| --- | --- | --- | --- | --- |
| Myh7 | Mouse | CAGAACACCAGCCTCATCAACCAG | TTCTCCTCTGCGTTCCTACACTCC |  |
| BNP | Mouse | TGCTGGAGCTGATAAGAGAAAA | GAAGGACTCTTTTTGGGTGTTC |  |
| TNF-α | Mouse | CCTGTAGCCCACGTCGTAG | GGGAGTAGACAAGGTACAACCC |  |
| IL-6 | Mouse | CTGCAAGAGACTTCCATCCAG | AGTGGTATAGACAGGTCTGTTGG |  |
| IL-1β | Mouse | TCGCAGCAGCACATCAACAAGAG | AGGTCCACGGGAAAGACACAGG |  |
| Bax | Mouse | CCGGCGAATTGGAGATGAACT | CCAGCCCATGATGGTTCTGAT |  |
| Bcl2 | Mouse | GATGACTTCTCTCGTCGCTAC | GAACTCAAAGAAGGCCACAATC |  |
| Caspase-3 | Mouse | TGGTGATGAAGGGGTCATTTATG | TTCGGCTTTCCAGTCAGACTC |  |
| TGF-β | Mouse | CCAGATCCTGTCCAAACTAAGG | CTCTTTAGCATAGTAGTCCGCT |  |
| Collagen I | Mouse | TAAGGGTCCCCAATGGTGAGA | GGGTCCCTCGACTCCTACAT |  |
| α-SMA | Mouse | CTATGCTCTGCCTCATGCCA | CTCACGCTCAGCAGTAGTCA |  |
| Nrf2 | Mouse | TCTTGGAGTAAGTCGAGAAGTGT | GTTGAAACTGAGCGAAAAAGGC |  |
| HO-1 | Mouse | AAGCCGAGAATGCTGAGTTCA | GCCGTGTAGATATGGTACAAGGA |  |
| NQO-1 | Mouse | AGGATGGGAGGTACTCGAATC | AGGCGTCCTTCCTTATATGCTA |  |
| UCP-2 | Mouse | CGGAGATACCAGAGCACTGTC | TGGCATTTCGGGCAACATTGG |  |
| β-actin | Mouse | CTACCTCATGAAGATCCTGACC | CACAGCTTCTCTTTGATGTCAC |  |
| Myh7 | Rat | CCAGAACACCAGCCTCATCAACC | CACCGCCTCCTCCACCTCTG |  |
| BNP | Rat | CTCCAGAACAATCCACGATG | ACAGCCCAAGCGACTGACT |  |
| TNF-α | Rat | TACTCCCAGGTTCTCTTCAAGG | GGAGGCTGACTTTCTCCTGGTA |  |
| IL-6 | Rat | ACTTCCAGCCAGTTGCCTTCTTG | TGGTCTGTTGTGGGTGGTATCCTC |  |
| IL-1β | Rat | CTCACAGCAGCATCTCGACAAGAG | TCCACGGGCAAGACATAGGTAGC |  |
| Bax | Rat | CCAGGACGCATCCACCAAGAAG | GCTGCCACACGGAAGAAGACC |  |
| Bcl2 | Rat | ACGGTGGTGGAGGAACTCTTCAG | GGTGTGCAGATGCCGGTTCAG |  |
| Caspase-3 | Rat | GCAGCAGCCTCAAATTGTTGACTA | TGCTCCGGCTCAAACCATC |  |
| TGF-β | Rat | CAAGGAGACGGAATACAGGGC | AGGAAGGGTCGGTTCATGTC |  |
| Collagen I | Rat | TGTTGGTCCTGCTGGCAAGAATG | GTCACCTTGTTCGCCTGTCTCAC |  |
| α-SMA | Rat | TGTGCTGGACTCTGGAGATG | GATCACCTGCCCATCAGG |  |
| Nrf2 | Rat | ACTGGATGAAGAGACCGGAGA | ATCCAGGGCAAGCGACTCAT |  |
| HO-1 | Rat | TCGACAACCCCACCAAGTTC | GAGGTAGTATCTTGAACCAGGCT |  |
| NQO-1 | Rat | TCCGAAGCATTTCAGGGTCG | TCTGCGTGGGCCAATACAAT |  |
| UCP-2 | Rat | TCTTCTGGGAGGTAGCAGGA | GCTCTGAGCCCTTGGTGTAG |  |
| β-actin | Rat | AAGTCCCTCACCCTCCCAAAAG | AAGCAATGCTGTCACCTTCCC |  |
